# The effects of cervical kinesiotaping on neck pain, range of motion, and disability in patients following thyroidectomy: a randomized, double-blind, sham-controlled clinical trial*

**DOI:** 10.3906/sag-1812-55

**Published:** 2019-08-08

**Authors:** Aysun GENÇ, Süleyman Utku ÇELİK, Volkan GENÇ**, Derya GÖKMEN, Birkan Sonel TUR

**Affiliations:** 1 Department of Physical Medicine and Rehabilitation, Faculty of Medicine, Ankara University, Ankara Turkey; 2 Clinic of General Surgery, Gülhane Training and Research Hospital, Ankara Turkey; 3 Department of General Surgery, Faculty of Medicine, Ankara University, Ankara Turkey; 4 Department of Biostatistics, Faculty of Medicine, Ankara University, Ankara Turkey

**Keywords:** Kinesiotaping, cervical spine, neck pain, range of motion, thyroidectomy, disability

## Abstract

**Background/aim:**

This was a randomized, double-blind, sham-controlled study.****Thyroidectomy is a frequently performed surgical procedure and head and neck extension during this operation facilitates surgery. Patients may experience postoperative neck pain and cervical range of motion (ROM) limitation due to the surgical position following thyroidectomy. It was aimed herein to investigate the short-term effects of kinesiotaping (KT) applied to the cervical spine on neck pain, ROM, and disability in patients following thyroidectomy.

**Materials and methods:**

A total of 74 patients were randomly assigned to be treated with either KT (Group 1, n = 37) or sham taping (Group 2, n = 37) using a computer-generated random number list. Neck pain, cervical ROM, and neck disability were evaluated with a visual analog scale (VAS), inclinometer, and the Neck Disability Index (NDI) questionnaire, respectively.

**Results:**

There were no significant differences with respect to age, sex, educational background, or body mass index between the groups.****While there were no significant differences with respect to improvement of the VAS and change of the ROM and NDI values between the groups, patients in Group 1 needed less paracetamol than patients in Group 2 (P = 0.011).

**Conclusion:**

This study showed that cervical KT application following thyroidectomy does not have a positive effect on neck pain, ROM, or disability, but nonetheless, it reduces analgesic consumption.

## 1. Introduction

Head and neck surgeries, such as thyroidectomy and parathyroidectomy, are some of the most common surgical operations. During these procedures, the patient is placed in the supine position with neck extension in order to provide better access to the gland and facilitate the surgical operation [1]. However, patients may experience postoperative posterior neck pain, occipital headaches, shoulder and neck movement difficulties, shoulder stiffness, and cervical range of motion (ROM) limitation due to the surgical position [2,3]. These symptoms may be observed for a long time after surgery and may even negatively impact the patients’ quality of life [2,4]. Moreover, some serious complications, such as bilateral hypoglossal palsy [5], cervical artery dissection [6], and tetraplegia [7] caused by neck hyperextension secondary to intubation or dental or thyroid surgery procedures, have been reported in the literature. The degree of neck extension and risk factors such as advanced age and the presence of spondylosis and/or spinal stenosis are considered by clinicians before medical procedures requiring prolonged neck hyperextension [1,8,9].

Although more than 80% of patients complain of posterior neck pain, headaches, and ROM limitations following thyroidectomy, the majority of studies have focused on pain originating from the incision site, and these symptoms have often been ignored [1,3]. Analgesics have been commonly administered and they are useful for the control of incisional pain [1,10,11], but there has been no direct evidence to show their effectiveness for discomfort symptoms such as posterior neck pain, neck movement difficulties, and cervical ROM limitation following head and neck surgeries [3]. Recently, a variety of treatment modalities have been used to overcome these disturbing symptoms, such as intraoperative transcutaneous electrical nerve stimulation (TENS) [1], preoperative bilateral greater occipital nerve (GON) block [3,11], bilateral superficial cervical plexus block combined with bilateral GON block [12], and postoperative neck stretching exercise [2,4]. 

Kinesiotaping (KT) was created in the mid-1970s by Dr Kenzo Kase (https://kinesiotaping.com/about/). It was initially used in the treatment of musculoskeletal disorders or the prevention of sport injuries, but it began to be used over time for other purposes, including hypertension, premenstrual syndrome, and constipation [13–15]. The application of KT improves blood and lymphatic circulation, reduces pain with analgesic system activation, increases ROM, and also supports muscular activities [16–19]. Herein, it was aimed to investigate the short-term effects of KT applied to the cervical spine on posterior neck pain, cervical ROM, and disability in patients following thyroidectomy. 

## 2. Materials and methods

### 2.1. Participants

A total of 80 patients were enrolled in this prospective, double-blind, randomized controlled study. The study was performed at the Ankara University Faculty of Medicine in Ankara, Turkey, between January 2017 and January 2018, with patients who underwent a total thyroidectomy. Patients who were younger than 18 years of age, had an open wound or cellulitis that would compromise KT application, or had a history of cervical surgery, diagnosis of cervical radiculopathy or myelopathy, skin hypersensitivity to tape material, or medical therapy including analgesics, opioids, and corticosteroids were excluded from the study. 

### 2.2. Collected data

The demographics, American Society of Anesthesiologists (ASA) physical status, body mass index (BMI), relevant comorbidities, and surgery duration were analyzed. As a routine procedure of the Department of Anesthesiology in our hospital, all of the patients were taken to the Post Anesthesia Care Unit (PACU) after surgery. Patients in the PACU were evaluated in terms of consciousness, mobility, breathing, circulation, color, and O2 saturation with the modified Aldrete score [20]. Scores of 9 and above indicate that the patient can be discharged from the PACU to their rooms. Neck pain was assessed using a visual analog scale (VAS, 0–10 cm) before surgery (BS) and 30 min, 4 h, 12 h, 24 h, and 7 days after discharge from the PACU. The ROM of the cervical spine in flexion, extension, lateral flexion, and right-left rotation were measured using a bubble inclinometer before and 24 h after the procedure. The inclinometer was placed in the sagittal and frontal plane on the head while the subject was seated for measurement of the flexion and extension ROM and the lateral flexion ROM, respectively. The right and left cervical rotation ROM was measured while the subject was in the supine position and the inclinometer was placed in the transverse plane on the forehead [21]. Moreover, disability was evaluated using the Turkish version of a validated 20-item Neck Disability Index (NDI; 0%–100%) before and 7 days after surgery (AS) [22].

### 2.3. Surgical procedure and positioning

Before the surgical procedure, the patient was positioned supine with a shoulder roll. The head was placed on a donut cushion and the neck was extended to provide maximal exposure [23]. Standardized surgical procedures were performed by the same surgeon (VG), who was not aware of patient group assignments. 

### 2.4. Kinesiotape application

Application of the KT (Kinesio Tex Gold, Tokyo, Japan) was performed as soon as the patients were taken into their room AS. It was applied to participants in Group 1 (experimental group) in accordance with the standardized protocol described by Kase et al. [24]. While the patient was seated, the 5-cm Y-shaped strip was placed symmetrically over the posterior cervical extensor muscles with a degree of tightness of 25% and placed from the dorsal region (T1–T2) to the upper-cervical region (C1–C2). Each tail of the bandage was attached to the skin so as to provide the cervical spine with contralateral flexion and rotation. The second strip was 5 cm wide and shaped like a capital I. It was applied perpendicular to the Y-strip, over the midcervical region (C3–C6), with the cervical spine in flexion to apply tension to the posterior structures. In Group 2 (sham group), only a 5-cm-wide I-shaped tape was placed over the midcervical region with the patient’s cervical spine in flexion to apply tension to the posterior structures without applying tension in the transverse plane. Tapes were administered by the same certified KT practitioner (BST), who masked the treatment group, and were replaced for the patients at the end of the first week. Whether there were side effects related to the KT was recorded. The assessment of pain with the VAS, ROM measurement, and NDI application were performed by the same researcher (AG), who was not aware of patient group assignments. 

All of the patients were discharged on the first postoperative day. The need for analgesics (paracetamol, a maximum daily dose of 1000 mg) was also recorded within the first 7 days after the operation.

### 2.5. Statistical analyses

All of the data were collected by a researcher (SUÇ) who was not aware of patient group assignments. Data were expressed as the mean (standard deviation) and range for continuous variables and frequency (percentage) for the categorical variables. The Shapiro–Wilk test was used to assess the normality assumption for the continuous variables. The differences in proportions between the groups were compared using chi-square or Fisher exact tests as appropriate. The Mann–Whitney U test was used to evaluate differences between the groups in terms of nonnormally distributed continuous variables. The Wilcoxon signed rank test was used to evaluate differences between, after, and before the measurements. P < 0.05 was considered statistically significant. All of the statistical data were analyzed using SPSS 16.0 (SPSS Inc., Chicago, IL, USA).

## 3. Results

**Figure 1:**
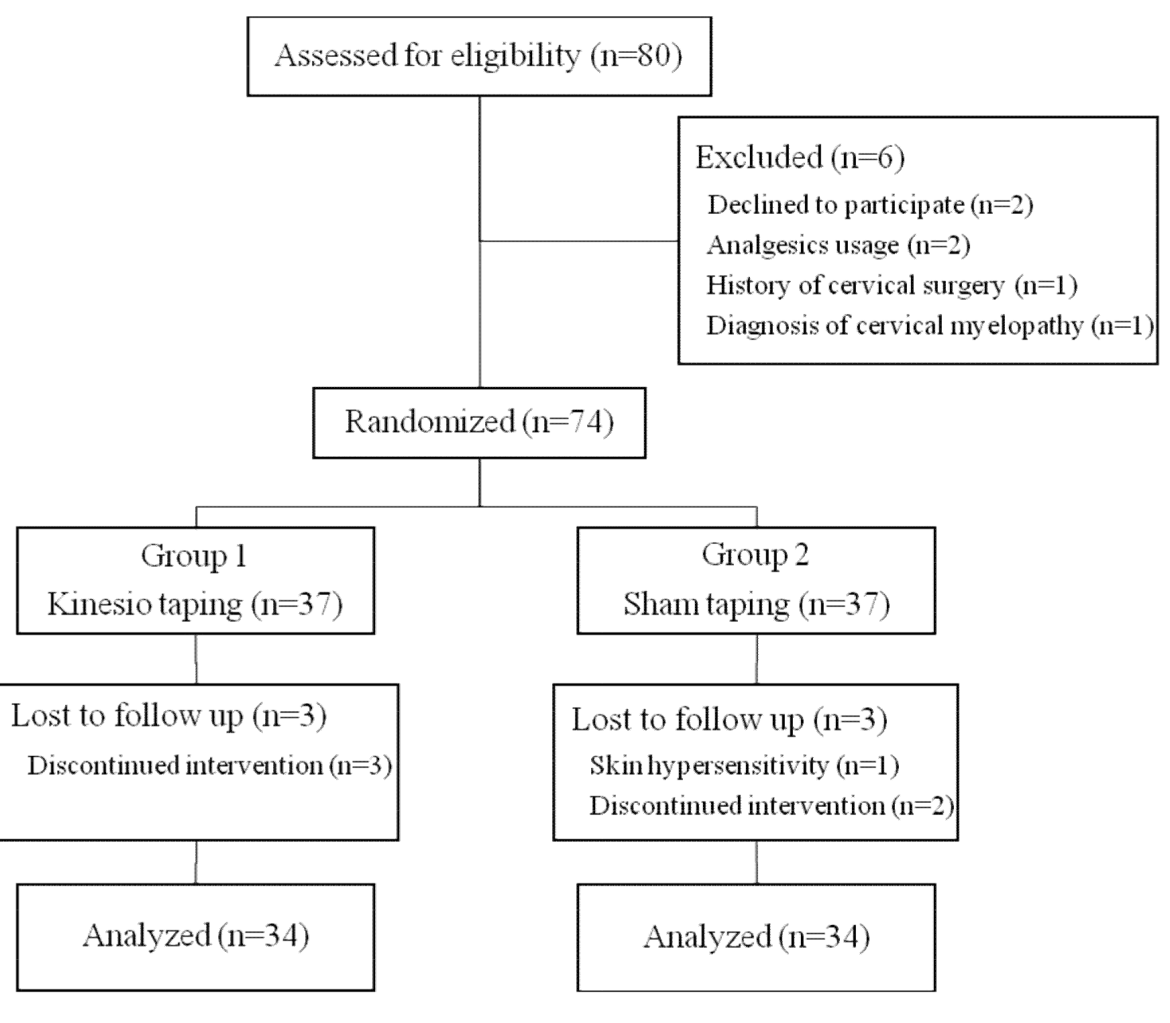
CONSORT flow diagram.

Six patients were excluded from the study and a total of 74 patients were randomly assigned to receive either KT (Group 1, n = 37) or sham taping (Group 2, n = 37) using a computer-generated random number list (Figure 1). From each group, 3 patients dropped out of the study and each group was completed with 34 patients. The participants, the surgeon, the researcher who administered the KT, and the researcher who evaluated the outcomes were all blinded as to the group allocations.

The mean ages****of the Group 1 and Group 2 participants were 51.6 ± 14.9 and 49.2 ± 16.3 years, respectively. Table 1 shows a comparison of the baseline demographics and clinical characteristics between the two groups. There was no significant difference with respect to age, sex, educational background, BMI, ASA score, or surgery duration.

**Table 1:** Demographics and the clinical characteristics of the two groups.

Variable	Group 1, n = 34	Group 2, n = 34
Age, years, mean ± SD	51.6 ± 14.9	49.2 ± 16.3
Sex, male / female	10 / 24	7 / 27
Educational background, n		
Primary school	14	10
Middle school	8	8
High school	6	9
University	6	7
BMI, kg/m2, mean ± SD	30.2 ± 5.8	28.3 ± 6.0
ASA physical status, I / II / III	22 / 11 / 1	20 / 10 /4
Surgery duration, min	113.1 ± 40.6	117.5 ± 30.0

BMI: Body mass index, ASA: American Society of Anesthesiologists.

**Figure 2:**
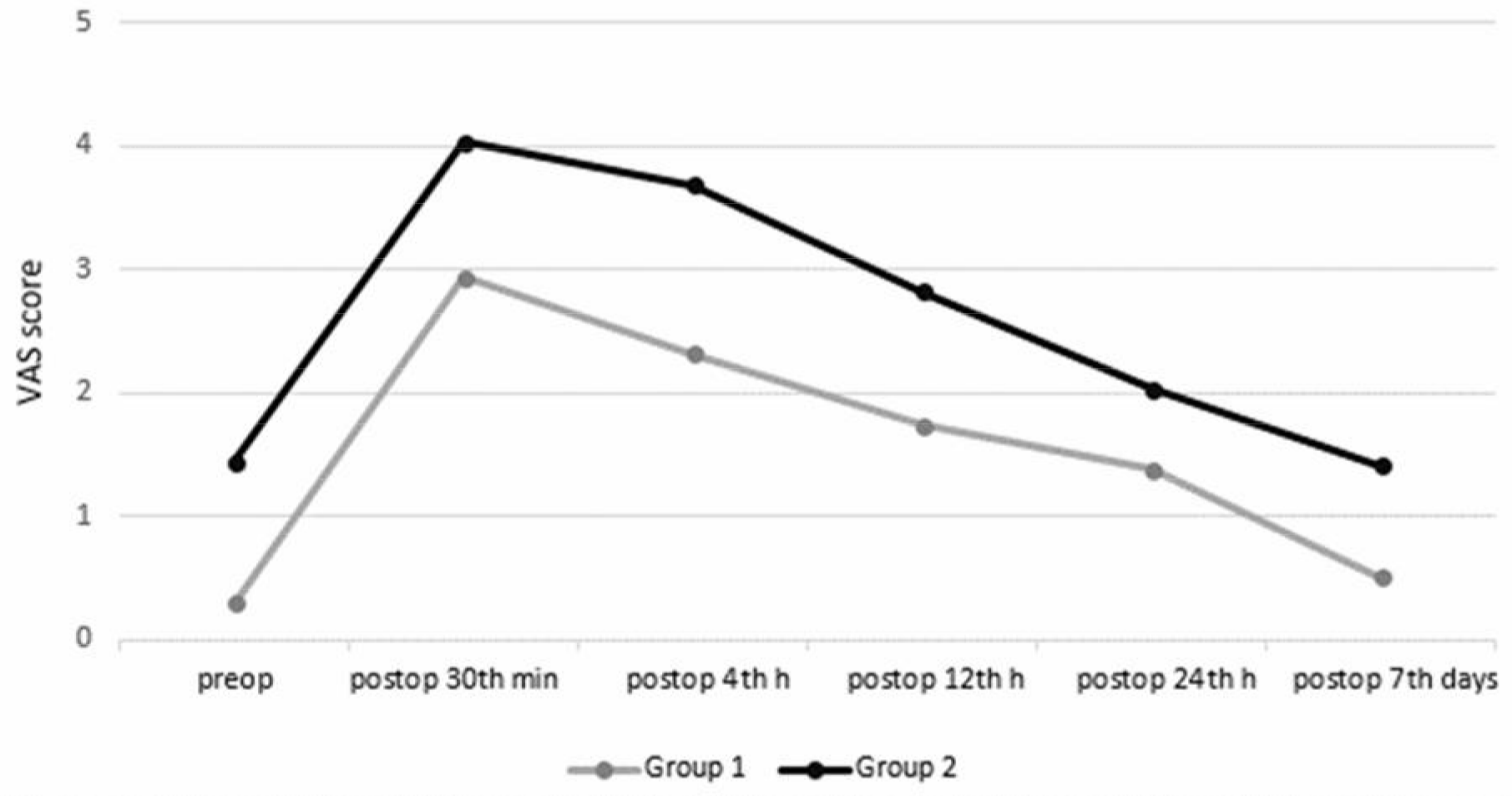
Change of the visual analog scale (VAS) in the groups.

In terms of the VAS, there was a significant difference between the groups in favor of Group 1 (P = 0.006). Moreover, there were significant time effects between the time points, except between BS and 7 days AS, 30 min, and 4 h (P < 0.001). However, interaction between group and time was not statistically significant (P = 0.838) (Figure 2). There was no significant difference with respect to the change of ROM values of the cervical spine in flexion, extension, right-left flexion, and right-left rotation between the groups during the study. Changes between the preoperative and postoperative 7th day of the NDI score were 0.6% in Group 1 and 3.1% in Group 2, but the differences were not statistically significant (P = 0.486) (Table 2). However, the mean requirement for the use of analgesics within the first 7 days after the operation was significantly (P = 0.011) less in Group 1 (1720 ± 1755 mg) than in Group 2 (2574 ± 1620 mg).

**Table 2:** Comparison of the VAS, ROM, and NDI values before and after surgery.

VAS (0–10)	BS	30 min	4 h	12 h	24 h	7 days	Overall	P1 (Pa, Pb, Pc)
Group 1	0.29 ± 0.87	2.94 ± 2.96	2.32 ± 2.67	1.74 ± 2.15	1.38 ± 1.74	0.50 ± 0.90	1.53±1.52	0.006, < 0.001, 0.838
Group 2	1.44 ± 2.06	4.03 ± 3.05	3.68 ± 3.01	2.82 ± 2.09	2.03 ± 1.94	1.41 ± 1.98	2.57±1.52	
Overall	0.87±1.58	3.49±3.01	3±2.84	2.28±2.12	1.71±1.85	0.96±1.56		
ROM	Extension	Flexion	Right lateral flexion	Left lateral flexion	Right rotation	Left rotation		
Group 1	33.14 ± 21.18	14.50 ± 20.19	15.47 ± 20.55	15.27 ± 18.50	22.32 ± 21.52	20.98 ± 18.59		
Group 2	26.00 ± 24.03	17.60 ± 16.78	13.40 ± 22.40	15.59 ± 22.66	19.56 ± 20.29	20.32 ± 20.08		
P2	0.152	0.510	0.871	0.474	0.662	0.417		
NDI (0%–100%)	BS	7th day	BS to 7th day					P3
Group 1	9.23 ± 6.66	9.87 ± 6.33	0.64 ± 7.14					0.486
Group 2	16.01 ± 14.44	19.14 ± 15.55	3.12 ± 14.51					

VAS: Visual analog scale, ROM: range of motion, NDI: neck disability index. Values are mean units ± SD. BS: Before surgery, AS: after surgery (30 min, 4 h, 12 h, 24 h, and 7 days). P1: Comparison of the changes from the BS values to the AS values between the 2 groups (Pa: comparison between groups, Pb: comparison between time points, Pc: interaction between group and time). P2: Comparison of the percentage changes of the ROM values from the BS values to the 24-h AS values between the 2 groups [((BS-AS)/ BS) × 100]. P3: Comparison of the percentage changes of the NDI% values from the BS values to the 7th day AS values between the 2 groups.

## 4. Discussion

We have demonstrated that cervical KT application following thyroidectomy did not have a positive effect on neck pain, ROM, or disability; it only reduced analgesic consumption. In the postoperative period, effective pain management is essential and the primary target is to improve postoperative comfort and satisfaction of the patient, facilitate recovery and functional ability, and promote rapid discharge from the hospital. To the best of our knowledge, this was the first study to evaluate the efficacy of KT on posterior neck pain after a surgery requiring cervical hyperextension. 

Occasionally, postthyroidectomy pain comes to mind as incisional pain. However, two types of pain have been expressed by patients following thyroidectomy. The first is incisional pain, while the other is positional posterior neck pain, caused by hyperextension applied to the patient’s neck during surgery. For the first type of pain, the patient localizes the pain at the incision site, while for the second type of pain, the patient defines pain in the back of the neck. Therefore, the type of pain can be easily distinguished after surgery. Both pain types usually occur immediately after surgery and last for a couple of days.

Surgical procedures that require neck hyperextension are performed frequently and although posterior neck pain and stiffness are seen at a very high rate, it is interesting to note that this issue has not been adequately discussed in the literature. In one of the limited studies that specifically addressed posterior neck pain after thyroidectomy, Han et al. reported that 80% of patients complained of this and showed that preoperative bilateral GON blocked with bupivacaine effectively reduced posterior neck pain and occipital headaches after thyroid surgery [3]. In another study, Takamura et al. found that stretching exercises effectively reduced postoperative neck symptoms, such as discomfort, and also reduced the use of analgesics following thyroidectomy [4]. A recent study by Park et al. reported that intraoperative TENS applied to the trapezius muscle reduced posterior neck pain after thyroidectomy [1]. To date, no published report has described the use of KT for decreasing analgesic consumption in patients undergoing thyroidectomy. 

In recent years, there has been an increasing amount of literature demonstrating that KT had a positive effect on pain and the prevention and treatment of joint injury, and provided excellent pain-free ROM in the musculoskeletal system. Thus, the use of KT has increased dramatically over the last decade [18,19,25]. However, the underlying mechanisms for the possible beneficial effects of KT on musculoskeletal pain remain unclear. It is thought that the application of KT improves blood and lymphatic circulation, reduces pain intensity with analgesic system activation, increases ROM, and also reduces muscle tension [16–19,26]. Kelle et al. found that KT provided significant improvements in pain and disability in patients with acute nonspecific low back pain [26]. In a randomized, double-blind, placebo-controlled study, Ay et al. analyzed the data from 61 patients and concluded that the application of cervical KT in cervical myofascial pain syndrome led to improvements in pain, the pressure pain threshold on the trigger point, ROM, and disability measures [27]. Hernandez et al. compared the effectiveness of cervical spine thrust manipulation and KT applied to the neck and revealed that KT was as efficacious as cervical thrust manipulation for decreasing neck pain and disability in patients with mechanical idiopathic neck pain [18].

The current findings observed in this study contradict previous studies examining the effect of KT on various musculoskeletal system conditions. In the current study, no significant difference was identified with regard to improvement of the ROM values of the cervical spine in flexion, extension, right-left flexion, and right-left rotation between the KT group and the sham taping group. In terms of neck disability, while the KT group revealed a similar NDI score when comparing baseline to day 7 AS (9.23% to 9.87%), the mean NDI score had deteriorated in the sham group (16.01% to 19.14%), but this difference was not statistically significant. Ay et al. reported positive effects of KT on the ROM values and NDI scores in patients with cervical myofascial syndrome, but these patients were treated with KT for 15 days [27]. Moreover, Takamura et al. followed the effects of stretching exercises on postoperative neck discomfort until the end of the first year after thyroid surgery [4]. We applied KT for only 7 days and evaluated ROM values and NDI scores in very short intervals. While the results of this study indicated no positive influence of KT on pain intensity, it did result in a reduced requirement for the use of analgesics within the first 7 days after the operation. Although these data appear to contradict each other, we think that analgesic consumption is not only related to pain, but also to unpleasant symptoms like feelings of stretching, choking, or pressing in the neck. Indeed, Takamura et al. [4] revealed this relationship in their study. However, these unpleasant symptoms were not evaluated in this study.

 A limitation of this study was that it did not have a no-treatment control group, which could draw comparison between the KT and sham taping groups. Therefore, the possibility of a placebo effect of the tapings cannot be excluded. Another limitation was that the study focused on only the short-term results of KT application. The key strength of the current study was that the data were prospectively collected and the study was designed as double-blinded and sham-controlled.

In conclusion, this study showed that cervical KT application following thyroidectomy did not have a positive effect on neck pain, ROM, or disability; it only reduced analgesic consumption. However, further prospective studies with larger numbers of subjects are needed to provide definitive evidence for this relationship.
